# Mitogen-activated protein kinases (MAPKs) are modulated during *Francisella tularensis* infection, but inhibition of extracellular-signal-regulated kinases (ERKs) is of limited therapeutic benefit

**DOI:** 10.1007/s10096-016-2754-1

**Published:** 2016-10-06

**Authors:** R. J. Saint, R. V. D’Elia, C. Bryant, G. C. Clark, H. S. Atkins

**Affiliations:** 1 CBR Division, Defence Science and Technology Laboratory, Porton Down, Salisbury, SP4 0JQ UK; 2 Department of Veterinary Medicine, University of Cambridge, Madingley Road, Cambridge, CB3 0ES UK; 3 University of Exeter, Exeter, UK

## Abstract

*Francisella tularensis* is a Gram-negative intracellular bacterium that causes the disease tularemia. The disease can be fatal if left untreated and there is currently no licenced vaccine available; the identification of new therapeutic targets is therefore required. Toll-like receptors represent an interesting target for therapeutic modulation due to their essential role in generating immune responses. In this study, we analysed the in vitro expression of the key mitogen-activated protein kinases (MAPKs) p38, JNK and ERK in murine alveolar macrophages during infection with *F. tularensis*. The phosphorylation profile of ERK highlighted its potential as a target for therapeutic modulation and subsequently the effect of ERK manipulation was measured in a lethal intranasal *F. tularensis* in vivo model of infection. The selective ERK1/2 inhibitor PD0325901 was administered orally to mice either pre- or post-challenge with *F. tularensis* strain LVS. Both treatment regimens selectively reduced ERK expression, but only the pre-exposure treatment produced decreased bacterial burden in the spleen and liver, which correlated with a significant reduction in the pro-inflammatory cytokines IFN-γ, MCP-1, IL-6, and TNF-α. However, no overall improvements in survival were observed for treated animals in this study. ERK may represent a useful therapeutic target where selective dampening of the immune response (to control the damaging pathology seen during infection) is combined with antibiotic treatment required to eradicate bacterial infection. This combination treatment strategy has been shown to be effective in other models of tularemia.

## Introduction

Toll-like receptors (TLRs) are sentinels of the innate immune system, functioning to detect invading microorganisms by binding to pathogen-associated molecular patterns (PAMPs) such as lipids, proteins and nucleic acids [1–3]. The binding event triggers an intracellular signalling cascade through dimerisation of the receptor and the subsequent recruitment of signalling proteins, leading to the production of immune mediators such as chemokines and cytokines. Eleven TLRs have been identified in humans located on multiple cell types including neutrophils, macrophages, and epithelial cells [4, 5]. TLR activity is vital for pathogen clearance, but dysregulation can occur which can have severe consequences for the host. Indeed, it is now well documented that an overactive immune response can be more damaging to the host than the progression of infection by the pathogen [6]. This fine balance is illustrated when a host is infected via the inhalational route with the Gram-negative facultative intracellular bacterium *Francisella tularensis*, where there is an initial suppression of pro-inflammatory cytokines followed by a “cytokine storm” prior to death [7].

The primary detection of *F. tularensis* was originally thought to be mediated by TLR4 [8, 9]. However, more recent studies demonstrate that it is likely that TLR2, and not TLR4, confers the ability to detect *F. tularensis* [10–12]. TLR4 was ruled out as a major receptor in the detection of *F. tularensis* as TLR4-defective mice were no more susceptible to an inhalational infection with *F. tularensis* than wild-type mice and the survival rates were similar in both mouse strains [13]. In contrast, several studies have reported a requirement for TLR2 in the detection of *F. tularensis* following in vivo infection by multiple challenge routes or in different types of cultured cell [10, 14–19]. More recently, the importance of TLR2-mediated priming of the inflammasome to intracellular survival has been highlighted [20]. Furthermore, a TLR2 deficiency enhanced murine susceptibility to pulmonary infection by the *F. tularensis* live vaccine strain (LVS) and resulted in decreased production of pro-inflammatory cytokines including TNF-α and IL-6 [10].

A number of studies have previously demonstrated the potential therapeutic benefits of specifically targeting TLRs for the treatment of infectious diseases including those caused by Francisella *sp*. For example, bacterial DNA containing un-methylated CpG motifs have been used to stimulate TLR9 and induce protection against subsequent challenge with the intracellular pathogens *F. tularensis* strain LVS and *Listeria monocytogenes* [21]. Furthermore, stimulation of TLR3 using the synthetic dsRNA analogue poly(I:C) has been evaluated for the treatment of *F. tularensis* strain LVS or strain SchuS4 infection in Balb/c mice [22]. Treatment as a monotherapy 1 h prior to or 1 h after intranasal infection led to significant improvements in the survival of mice, correlating with an increased influx of neutrophils to the lungs and early up-regulation of cytokines IL-6, MCP-1, and TNF-α [22]. However, it is noteworthy that a combination therapy approach with the antibiotic levofloxacin was required for full recovery.

In comparison to TLR receptors, less research has focused on understanding the activation and phosphorylation states of the downstream immune signalling cascade proteins during *F. tularensis* infection and/or the potential for selectively targeting these pathways with therapeutics. Previously, it has been demonstrated that the up-regulation of p38, IkB and c-Jun occurs following infection of murine macrophages with *F. tularensis* strain LVS [15]. In addition, it has been demonstrated that the uptake of *Francisella novicida* by macrophages is dependent upon ERK activation, and modulation of the MAPK pathways is important for intracellular survival of *F. tularensis* LVS [23, 24]. Furthermore, within an in-vivo setting it was found that the inhibition of GSK3B (a serine/threonine protein kinase) with lithium chloride significantly reduces the production of pro-inflammatory cytokines such as IFN-γ, IL-12p40, and TNF-α in *F. tularensis* LVS-infected mice, and increases survival from 60 % in untreated mice to 90 % following treatment [25]. It is hypothesised that targeting specific proteins within the TLR signalling cascades may prevent the wide-ranging effects of direct TLR stimulation. In this study we sought to determine the in vitro expression profile of key mitogen-activated protein kinases (MAPKs) p38, JNK, and ERK following *F. tularensis* infection. Additionally, we determined the effect of inhibiting ERK expression in vitro and in vivo during a *F. tularensis* infection.

## Materials and methods

### Preparation of bacteria

*Francisella tularensis* live vaccine strain (LVS) was grown overnight on blood cysteine glucose agar (BCGA) plates incubated at 37 °C, and used to inoculate 10 ml PBS to an OD_600_ of 0.15 (∼1 × 10^9^ CFU/ml). The resulting bacterial suspension was diluted to the required inoculum. To confirm the CFU/ml of the inoculum, serial dilutions were cultured on BCGA plates for subsequent bacterial enumeration.

### Infection of MH-S cell line with *F. tularensis* LVS

A continuous cell line of murine alveolar macrophages (MH-S cells, ECACC UK) were seeded into 6-well plates (Corning, US) at ∼4 × 10^5^ cells/ml and incubated overnight at 37 °C to allow the cells to adhere. Supernatants were then removed and replaced with 1.5 ml of *F. tularensis* LVS inoculum to generate an MOI of 100. Plates were incubated at 37 °C for 2 h to allow for internalisation of the bacteria, and then the supernatants were removed and replaced with 1.5 ml fresh media. The plates were incubated at 37 °C and, at a range of timepoints, supernatants were removed and stored at −20 °C for cytokine analysis. The cells were washed once with chilled PBS and then lysed by adding 350 μl PhosphoSafe™ extraction reagent (Merck, US) before incubating on ice for 5 min. Full cell recovery was ensured by scraping wells with a rubber policeman (Corning, US): 100 μl of the lysate was used for bacterial enumeration, and a further 250 μl was centrifuged at 13,000 rpm for 5 min to remove cell debris before being stored at −20°C for analysis by western blotting.

### In-vivo infection and treatment with ERK inhibitor PD0325901

A total of 65 female 6- to 8-week-old Balb/c mice (Charles River Laboratories, UK) were separated into four groups: group I (20 mice), group II (20 mice), group III (15 mice) and group IV (10 mice). All mice, except those in group IV, were treated daily with either PBS or 0.05 μM PD0325901 (InvivoGen, France) by oral-gavage in a volume of 100 μl. Groups I and II were treated from 24 h pre-infection, while group III was treated from 48 h post-infection. The mice were infected via the intranasal route with approximately 2.5 × 10^4^ CFU of *F. tularensis* strain LVS. Scoring of the mice occurred twice daily throughout the study. At 48 h and 96 h post-challenge, five mice from each of groups I, II, III, and IV were culled and the lungs, liver, and spleen were removed and placed into aliquots of 2 ml PBS. Ten mice from each of groups I, II, III, and IV were monitored for changes in survival. Organs were homogenised though a 0.2 μm cell sieve (BD Biosciences) and screened for the levels of cytokines, activation of signalling proteins and bacterial enumeration on agar. All animal studies were carried out in accordance with the UK Scientific Procedures Act (1986). Animal handling and infection procedures were carried out by trained staff at Dstl.

### Western blots

Proteins were separated on a 4-20 % tris-glycine polyacrylamide gel and then transferred onto Invitrolon™ polyvinylidene fluoride (PVDF) membranes (Invitrogen) using a Novex® Semi-Dry Blotter (Invitrogen) according to manufacturer’s instructions. The PVDF membranes were then incubated in blocking buffer (5 % [w/v] skimmed milk powder and 0.1 % Tween® 20 (Sigma-Aldrich) dissolved in PBS) for 2 h at room temperature. To detect ERK activation, the membranes were washed in wash buffer (0.1 % Tween® 20 in PBS) before being incubated overnight at 4 °C with the primary antibody [p38 - #9218; P-p38 - #9211; SAPK/JNK - #9252; P-SAPK/JNK - #9251; ERK-1 - #4372; ERK-2 - #9108; P-ERK-1/2 - #9101 (NEB)] at a dilution of 1:1,000 in blocking buffer. The membranes were then rinsed in wash buffer and incubated for 1 h at room temperature with the secondary antibody [goat anti-rabbit IgG conjugated to horseradish peroxidise (Bio-Rad)] at a dilution of 1:3,000 in blocking buffer. The membranes were rinsed in wash buffer and developed using enhanced chemiluminescence (ECL)-Plus detection reagents (GE Healthcare) according to manufacturer’s instructions. The membranes were placed in a film cassette and exposed to ECL hyperfilm (GE Healthcare) which was allowed to air-dry before analysis by densitometry.

### Cytometric bead array

The quantities of the cytokines IFN-γ, MCP-1, IL-6, IL-12p70, IL-10, and TNF-α present within the lungs were measured using BD™ cytometric bead array (CBA) technology (BD Biosciences) on a BD™ FACScan flow cytometer (BD Biosciences) according to manufacturer’s instructions. A standard curve was generated and the concentration of each cytokine was interpolated using GraphPad Prism® 5.01 software (GraphPad). The levels of IL-12p70 and IL-10 were too low to be detected, and so only the results of IFN-γ, MCP-1, IL-6, and TNF-α are shown.

### Statistical analysis

All transformations of data and statistical tests were performed using GraphPad Prism® 5.01 software. Data from cytometric bead arrays were analysed using PRISM, by fitting a quadratic regression to the standard curves and reading the samples as unknowns.

## Results

ERK, p38, and JNK represent key signalling molecules in the MAPK cascade [26]. Activation of these proteins, by phosphorylation, has diverse biological effects including cell proliferation, apoptosis, and inflammation [27]. In this study, temporal changes in the expression of total ERK, p38, and JNK and the phosphorylated forms of the proteins produced by in vitro cultured alveolar macrophage (MH-S) cell line in response to infection with *F. tularensis* LVS was measured via western blot.

**Fig. 1:**
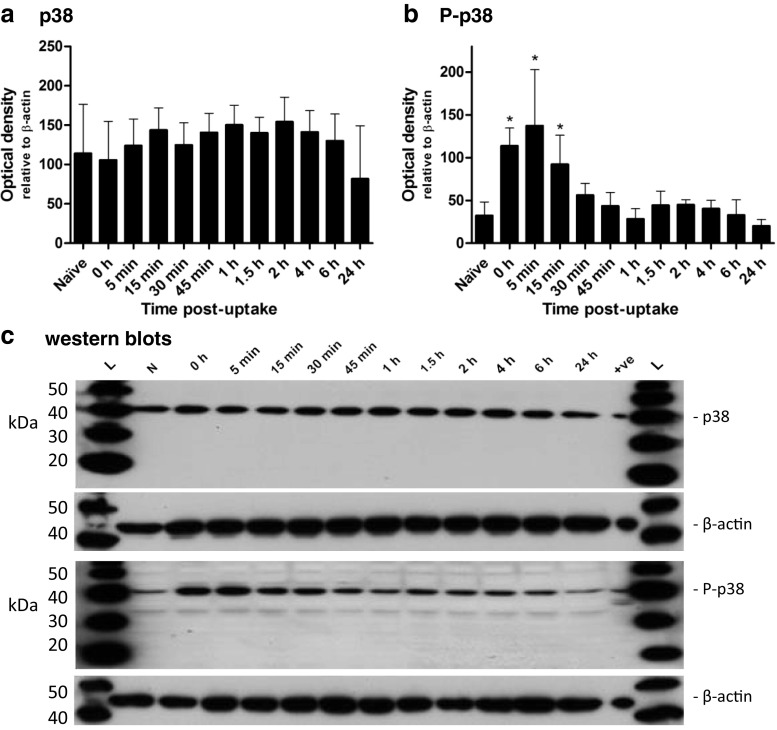
The expression and the levels of activated p38 in MH-S cells throughout the course of a *F. tularensis* LVS infection. The expression of p38 (**a**) and the levels of activated p38 (**b**) were determined at several time points after the 2-h uptake period. Representative western blots are shown below the corresponding graphs. The experiment was performed in triplicate (*n* = 3) and each replicate was performed in duplicate. *Error bars* show the 95 % confidence interval. Significant difference from naïve levels was determined by one-way ANOVA and a Dunnett’s multiple comparison post-test (* = *p* < 0.05). Western blot image: *L* — MagicMark™ ladder; *N* — naïve; *+ve* — positive control

**Fig. 2:**
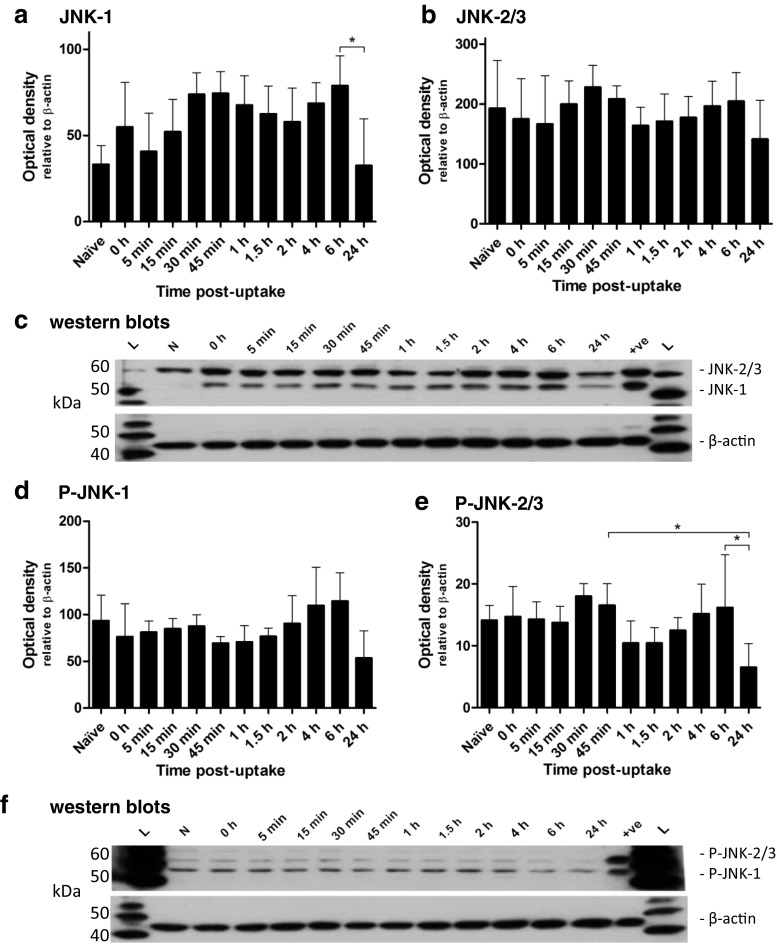
The expression and the levels of activated JNK-1 and JNK-2/3 in MH-S cells throughout the course of a *F. tularensis* LVS infection. The expression of JNK-1 (**a**) and JNK-2/3 (**b**) and the levels of activated JNK-1 (**c**) and JNK-2/3 (**d**) were determined at several time points after the 2-h uptake period. Representative western blots are shown below the corresponding graphs. The experiment was performed in triplicate (*n* = 3) and each replicate was performed in duplicate. *Error bars* show the 95 % confidence interval. Significant difference from naïve levels was determined by one-way ANOVA and a Dunnett’s multiple comparison post-test (* = *p* < 0.05). Western blot image: *L* — MagicMark™ ladder; *N* — naïve; *+ve* — positive control

The in vitro expression of p38 remained constant during the infection (Fig. 1a). However, there was a significant change in the p38 activation profile. First, a rapid increase in activation occurred immediately after the uptake of LVS; this was a transient response, and the quantities of activated protein present returned to naïve levels by 0.5 h (Fig. 1b). After the rapid and transient activation of p38 there was no re-activation measured throughout the rest of the infection assay. In comparison, limited in vitro activation of JNK proteins was observed during the experiment, with the exception of the 24-h time point, where there was a decrease in the presence of activated JNK-2/3 compared to that found at both 0.75 h and 6 h (Fig. 2d). The expression of total JNK-2/3 remained relatively constant (Fig. 2b), although there was a slight decrease in the expression of total JNK-1 from 6 to 24 h post-uptake (Fig. 2a).

**Fig. 3:**
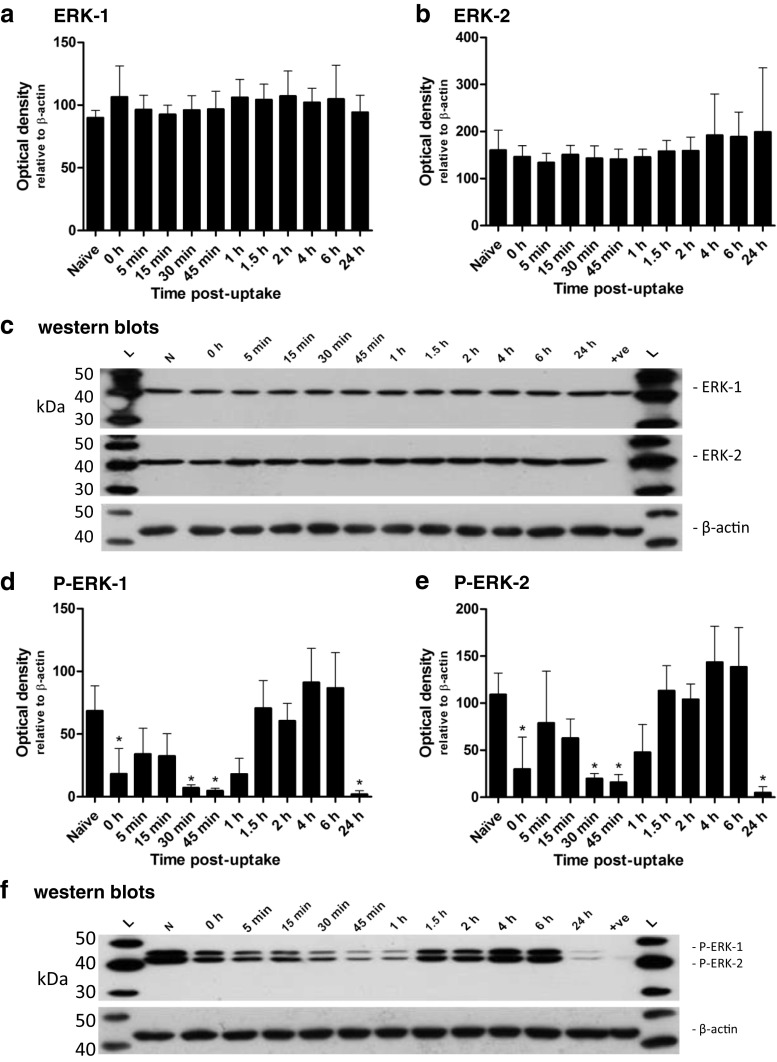
The expression and levels of activated ERK-1/2 in MH-S cells throughout the course of a *F. tularensis* LVS infection. The expression of ERK-1 (**a**) and ERK-2 (**b**) and the levels of activated ERK-1 (**c**) and ERK-2 (**d**) were determined at several time points after the 2-h uptake period. Representative western blots are shown below the corresponding graphs. The experiment was performed in triplicate (*n *= 3) and each replicate was performed in duplicate. *Error bars* show the 95 % confidence interval. Significant difference from naïve levels was determined by one-way ANOVA and a Dunnett’s multiple comparison post-test (* = *p *< 0.05). Western blot image: *L* — MagicMark™ ladder; *N* — naïve; *+ve* — positive control

The in vitro expression of total ERK-1 and ERK-2 did not alter significantly at any time point during the infection (Fig. 3a, b). The activation profiles of the two proteins did, however, display some interesting features (Fig. 3c, d). There appeared to be two phases of protein activation, particularly for ERK-2 (Fig. 3d). In the first hour after uptake a transient activation was observed. However, from 1.5 h there was a sustained activation of both ERK proteins which was maintained through to 6 h.

The interesting phosphorylation profile of ERK1/2 following *F. tularensis* LVS infection in vitro led us to consider modulation of the ERK proteins as an approach for treating infection. An inhibitor of ERK PD0325901was identified and assessed in vivo for its potential as a novel therapy. PD0325901 is a small molecule inhibitor with specific activity against phospho-ERK1/2 and increased potency compared to its predecessors [28, 29]. First, we demonstrated that PD0325901 was able to inhibit ERK expression during an in vitro *F. tularensis* macrophage infection assay and that it had a biological effect by significantly reducing the secretion of TNF-α (data not shown).

**Fig. 4:**
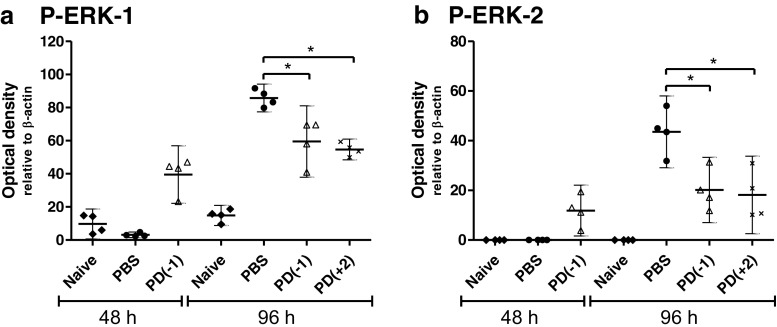
The effect of PD0325901 treatment (0.05 μM) on the activation of ERK-1 and ERK-2 in the lungs of *F. tularensis* LVS infected Balb/c mice. Groups of four mice infected via the intranasal route with 2.5 × 10^4^ CFU *F. tularensis* LVS were treated daily with PBS from 24 h pre-challenge (PBS), PD0325901 from 24 h pre-challenge [PD(−1)] or from 48 h post-challenge [PD(+2)]. Naive mice were used as a control. Detection of phosphorylated ERK-1 and ERK-2 in the lung homogenates of Balb/c mice was carried out by western blotting. Significant difference from PBS-treated group was determined using a one-tailed, unpaired *t*-test (* = *p *< 0.05). *Error bars* show 95 % confidence intervals

Subsequently, the potential for ERK inhibition (using PD0325901) was explored in vivo using a *F. tularensis* LVS intranasal model of infection. Balb/C mice infected with *F. tularensis* LVS showed a substantial increase in the level of ERK activation between 48 h and 96 h, indicating the involvement of ERK phosphorylation during infection in vivo, similarly to in vitro. Daily treatment of infected mice with PD0325901 significantly reduced both ERK-1 and ERK-2 activation in the lungs compared to PBS-treated mice at 96 h (Fig. 4a, b). This was found for both the PD0325901 treatment groups, irrespective of whether administration began at 24 h pre-infection or at 48 h post-infection. However, unexpectedly, at 48 h PD0325901-treated mice appeared to show an increase in ERK activation when compared to PBS-treated mice.

**Fig. 5:**
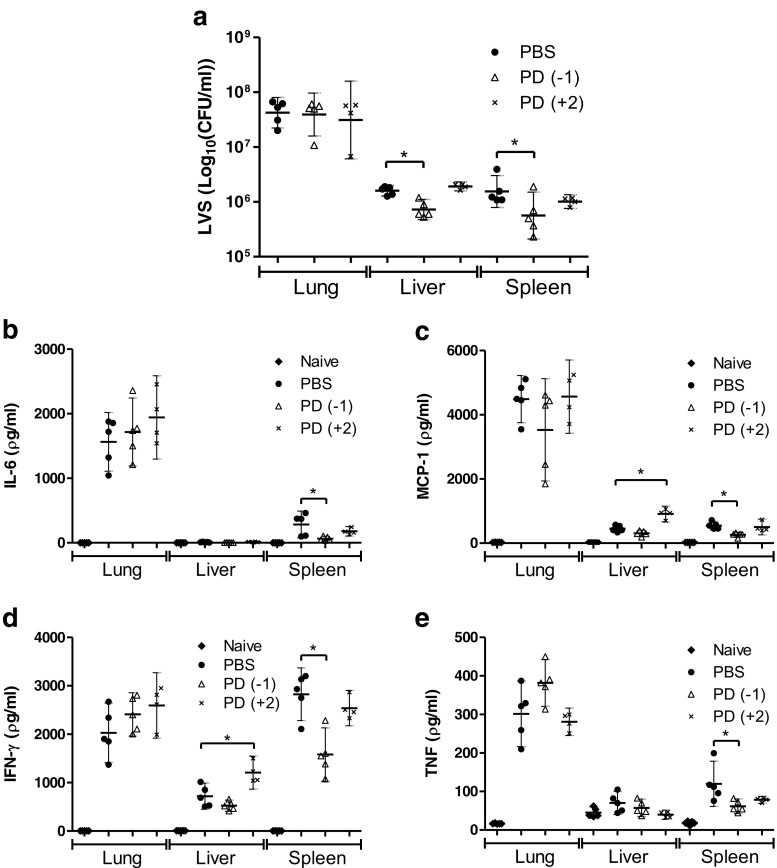
The effect of PD0325901 treatment (0.05 μM) on bacterial burdens and cytokine secretion in the organs of *F. tularensis* LVS-infected Balb/c mice. Groups of four mice infected via the intranasal route with 2.5 × 10^4^ CFU *F. tularensis* LVS were treated daily with PBS from 24 h pre-challenge (PBS), PD0325901 from 24 h pre-challenge [PD(−1)]or from 48 h post-challenge [PD(+2)]. Naive mice were used as a control. On day 4 post-challenge the organs were removed, homogenised and bacterial burdens were determined (**a**). The homogenates were also screened for the levels of IL-6 (**b**), MCP-1 (**c**), IFN-γ (**d**), and TNF-α (**e**). Significant difference from PBS-treated group was determined using a one-tailed, unpaired *t*-test (* = *p* < 0.05). *Error bars* show 95 % confidence intervals

To examine the effect of ERK inhibition on bacterial loads in the organs of infected mice, bacterial enumeration was carried out on samples taken from the lungs, liver, and spleens at 96 h post-infection (Fig. 5a). There was no significant difference in the numbers of bacteria present in the lungs of either of the PD0325901-treated groups compared to PBS-treated controls. However, there was a significant reduction in bacterial burden in the liver and spleen of mice treated with PD0325901 prior to infection when compared to PBS-treated control mice. These differences in the spleen and liver were not detected in mice treated with PD0325901 at 48 h post-infection.

**Fig. 6:**
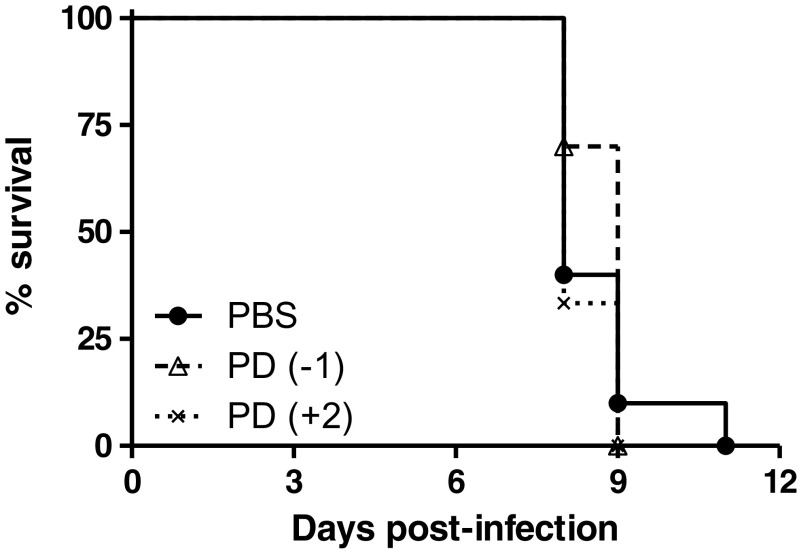
The effect of PD0325901 treatment (0.05 μM) on survival of Balb/c mice infected with *F. tularensis* LVS. Groups of four mice infected via the intranasal route with 2.5 × 10^4^ CFU *F. tularensis* LVS were treated daily with PBS from 24 h pre-challenge (PBS), PD0325901 from 24 h pre-challenge [PD(−1)] or from 48 h post-challenge [PD(+2)]. Naive mice were used as a control. The animals were scored daily and were culled upon reaching the humane endpoint

The effect of ERK inhibition on the inflammatory response was examined by measuring the secretion of downstream inflammatory cytokines in the lungs, liver, and spleen of naïve, PBS-treated or PD0325901-treated mice at 96 h after infection (Fig. 5b-e). Measurable levels of IL-6 (Fig. 5b) MCP-1 (Fig. 5c), IFN-γ (Fig. 5d) and TNF-α (Fig. 5e) were detected, but levels of IL-12p40 or IL-10 were below the limit of detection in all samples. At 96 h, no reduction in secretion of any of the cytokines was found in the lungs, despite the observed inhibition of ERK activation in the lungs of mice (Fig. 4). Mice treated from 48 h post-infection with PD0325901 displayed a significant increase in IFN-γ and MCP-1 in livers when compared to PBS-treated mice. The greatest effect of ERK inhibition was observed in spleen samples, where there was a significant decrease in the secreted levels of IFN-γ, MCP-1, IL-6, and TNF-α in mice treated with PD0325901 prior to infection (at day −1) compared to PBS-treated controls. This difference in the spleen was not seen when PD0325901 was administered at 48 h post-infection. Despite the PD0325901-associated inhibition of ERK activation within the lungs and the reduced bacterial burdens in the spleens and livers of infected mice, there was no significant difference in survival of PD0325901-treated groups compared to the PBS-treated groups (Fig. 6).

## Discussion

Antibiotics remain the most successful therapeutic regimen for *F. tularensis* infection, although efficacy is dependent on the correct administration of antibiotic in a timely manner [30]. Aiding antibiotic efficiency through immune-modulation is a strategy that is being explored for infectious diseases.

In these studies, we have demonstrated that total expression of the key MAPKs p38, JNK, and ERK were not significantly altered in an in vitro macrophage model of *F. tularensis* LVS infection. However, clear temporal changes in the phosphorylated states of the MAPKs were identified. Notably, the in vitro phosphorylation profile of ERK occurred in two distinct peaks of activation within infected macrophages. In addition, we identified that ERK was also significantly upregulated in vivo following intranasal infection of mice with *F. tularensis* LVS. By administering an inhibitor of ERK phosphorylation commencing both pre- and post-challenge, we aimed to evaluate this as a therapeutic strategy for treating infections caused by this organism. Following oral administration of the inhibitor to mice, a reduction of ERK phosphorylation was successfully achieved in vivo, with the greatest reductions observed when the compound was given prior to infection of mice. Treatment with the inhibitor resulted in significant decreases in bacterial burden in the spleen and liver but not at primary foci of infection of the lung. The reduction in bacterial burden correlated with a significant decrease in the pro-inflammatory cytokines IFN-γ, MCP-1, IL-6, and TNF-α, although survival of infected mice was not improved through administration of the inhibitor.

It has previously been reported that the activation of ERK signalling occurs in response to *F. tularensis* infection in vitro and in vivo [9, 19, 31]. Additionally, in vitro studies have demonstrated that MAPK activation, including ERK activation, plays an important role in *F. tularensis* LVS infection-induced apoptosis [32, 33]. To our knowledge, however, this is the first assessment of manipulation of ERK as a possible strategy for controlling *F. tularensis* infection. We found that ERK signalling in the lungs of infected mice could be successfully dampened by PD0325901 administration via oral gavage. This did not result in a reduction in bacterial numbers in the lungs (the site of bacterial infection), but there were significantly reduced bacterial burdens in both the liver and spleen of pre-treated mice. This may indicate that the proliferation of the bacteria from the primary site of infection has been reduced.

Sustained ERK activity promotes either intrinsic or extrinsic apoptotic pathways by induction of mitochondrial Cytochrome C release or caspase-8 activation, permanent cell cycle arrest, or autophagic vacuolisation [34]. Indeed, it has been suggested that the rapid proliferation of Francisella coincides with the initial activation of the apoptotic signalling pathway [35]. Therefore the relationship between ERK activation and the host mechanism of apoptosis could explain the observed differences in bacterial burden and cytokine response [33, 36]. In untreated *F. tularensis* infection (and in this study, in PBS-treated mice), the replication of bacteria within the lungs may induce apoptosis of infected cells such as alveolar macrophages via ERK activation, releasing the bacteria for proliferation throughout the host. In contrast, by limiting the activation of ERK through the administration of the inhibitor PD0325901, it is conceivable that the degree of apoptosis could be reduced, preventing cellular release and dissemination of the bacteria. In this context, the lungs act as a trap, with the result that bacterial burdens of this organ are unaffected whereas secondary sites, such as the liver and spleen, demonstrate reduced bacterial burdens as observed here. Further research involving the in vivo administration for the inhibitor and analysis of the presence/absence of apoptotic markers on cellular surfaces is required in order to fully evaluate this hypothesis, which was beyond the scope of this study.

In this study, ERK inhibition did not only alter bacterial burden but also had a significant effect on the pro-inflammatory secretion profile; specifically it was found that IFN-γ, MCP-1, IL-6, and TNF-α were all reduced in vivo following pre-treatment with PD0325901. It is well documented that an inflammatory response is essential for pathogen clearance, although it is also clear that pro-inflammatory responses contribute to the tissue damage and pathology seen during infection [6]. Indeed, the overactive immune response seen late during Francisella infection has been hypothesised to be one of the main contributors of detrimental outcome [7, 37]. Therefore, in this context, the modulation of the immune response achieved through inhibition of ERK with PD0325901 could be beneficial to survival of the host following infection. However, in this study we demonstrated that although dampening of ERK signalling was observed, along with an associated reduction in cytokine secretion and a decreased systemic proliferation of *F. tularensis* in treated mice, ultimately there was no measurable improvement in survival when compared to PBS-treated mice. This indicates that ERK inhibition alone is not sufficient to improve the outcome of infection with this pathogen. However, it is possible that a combination therapy, for example administering an ERK inhibitor with an antibiotic, could demonstrate a more beneficial outcome of infection. The ERK inhibitor could be used in this context to indirectly reduce systemic spread, whilst a bactericidal antibiotic directly kills the bacteria that remain within the lung. An analogous therapeutic strategy has already been shown to be beneficial in our laboratory for the treatment of the fully virulent SCHUS4 strain of *F. tularensis*, whereby inhibition of HMGB1, combined with levofloxacin use, significantly increased survival of infected mice compared to controls [7]. In addition, related approaches involving the administration of an antibiotic and an immunodulating therapy have been reported for other bacterial pathogens as well as in the treatment of sepsis [38]. It is important to note that the data generated in this study is derived from a murine model of tularemia as the availability of clinical samples from human cases of the disease is very limited, and it is not possible to conduct clinical trials in humans. However, it is well documented that there are similarities between human tularemia and disease presentation within mouse models, which are well accepted for research on *F. tularensis* [39, 40]. Therefore it is reasonable to extrapolate that targeting highly conserved MAPKs in humans would likely have a similar therapeutic benefit.

In conclusion, we have shown that PD0325901 can successfully alter ERK activation during an in vivo *F. tularensis* infection. It caused a reduction in bacterial burden in the liver and spleen if given prior to infection, and also significantly altered the host cytokine profile. The observed difference between mice given PD0325901 prior to or after infection is probably due to the time the compound requires to take effect in vivo and/or as a consequence of the bioavailability of the compound. Ultimately, therapeutic manipulation of ERK did not result in increased survival; however, it may be plausible that PD0325901 and/or other ERK inhibitors would be suitable for assessment within models of infection in combination with an anti-microbial therapy.
